# Attachment Representation Moderates the Influence of Emotional Context on Information Processing

**DOI:** 10.3389/fnhum.2016.00278

**Published:** 2016-06-08

**Authors:** Rainer Leyh, Christine Heinisch, Melanie T. Kungl, Gottfried Spangler

**Affiliations:** Institute of Psychology, Friedrich-Alexander-University Erlangen-NurembergErlangen, Germany

**Keywords:** attachment, emotion, P3, ERP, EEG, IAPS

## Abstract

The induction of emotional states has repeatedly been shown to affect cognitive processing capacities. At a neurophysiological level, P3 amplitude responses that are associated with attention allocation have been found to be reduced to task-relevant stimuli during emotional conditions as compared to neutral conditions suggesting a draining impact of emotion on cognitive resources. Attachment theory claims that how individuals regulate their emotions is guided by an internal working model (IWM) of attachment that has formed early in life. While securely attached individuals are capable of freely evaluating their emotions insecurely attached ones tend to either suppress or heighten the emotional experience in a regulatory effort. To explore how attachment quality moderates the impact of emotional contexts on information processing event-related potentials (ERPs) in 41 individuals were assessed. Subjects were instructed to detect neutral target letters within an oddball paradigm. Various images taken from the International Affective Picture System (IAPS) served as background pictures and represented negative, positive and neutral task-irrelevant contexts. Attachment representation was assessed using the Adult Attachment Interview (AAI) and individuals were assigned to one of three categories (secure, insecure-dismissing, insecure-preoccupied). At a behavioral level, the study revealed that negative emotionally conditions were associated with the detection of less target stimuli in insecure-dismissing subjects. Accordingly, ERPs yielded reduced P3 amplitudes in insecure-dismissing subjects when given a negative emotional context. We interpret these findings in terms of less sufficient emotion regulation strategies in insecure-dismissing subjects at the cost of accurate behavioral performance. The study suggests that attachment representation differentially moderates the relationship between emotional contexts and information processing most evident in insecure-dismissing subjects.

## Introduction

The first central key for the development into a social human being capable to interact properly with others is the attachment formed between a child and his or her primary caregiver (Sroufe, 1988). The caregiver is obliged to convey security and protection, and invite for exploration. Nevertheless, caregivers differ in their ability to handle infant emotions, what is most obvious in situations eliciting distress or anxiety. Parental sensitivity provides a developmental context for the child in which felt security is reliably balanced through the attachment figure (Ainsworth et al., 1978). Attachment theory postulates that these dyadic experiences of emotion regulation become internalized with time, resulting in internal working models (IWMs) of attachment (Bretherton and Munholland, 2008), which can be assigned to three prototypical patterns. Securely attached individuals have IWMs of their caregiver as available and responsive to their emotional needs and of the self as being worthy of love. In contrast, insecurely attached individuals have IWMs of their attachment figure as unavailable (insecure-dismissing) or unpredictable in behavior (insecure-insecure-preoccupied) and the self as being unworthy of love. IWMs resulting from different experiences are assumed to be carried over into adulthood and influence the way individuals organize their feelings, thoughts and behaviors in attachment-relevant situations (e.g., Bowlby, 1969) and especially how they handle their emotions (Cassidy, 1994). In particular, it has been theorized that securely attached individuals have free access to both negative and positive emotions. They are able to perceive and express their emotional state, which allows them a flexible evaluation and regulation within an emotional context. In contrast, insecurely attached individuals show deficits in their emotion regulation capacities. According to attachment theory insecure-dismissing subjects tend to hypo-activate emotional states as they have experienced rejection to expressed negative emotions earlier in life. Finally, based on the experience of a caregiver who only inconsistently responded to their psychological needs, insecure-preoccupied subjects are easily over-whelmed by negative emotions that—once activated—tend to dominate their mental state. The association between attachment security and a flexible regulation of emotions has been well established, including contexts that are not directly linked to the original attachment dyad (e.g., Kobak and Sceery, 1988; Zimmermann et al., 2001). In addition, attachment-related differences in emotional processing have been found to be evident on subconscious levels measured by biological markers such as endocrinological (e.g., Spangler and Grossmann, 1993), neurophysiological (e.g., Dawson et al., 2001) or electromyographic (e.g., Spangler et al., 2010) responses.

While during infancy and early childhood individual differences in IWMs of attachment are assessed by standardized observation of attachment behavior towards the caregiver (e.g., in the strange situation; Ainsworth et al., 1978), in adulthood they are assessed on a representational level. This can be done explicitly by the use of self-report questionnaires (e.g., Fraley et al., 2000) or implicitly by the use of the Adult Attachment Interview (AAI; George et al., 1985) or the Adult Attachment Projective (AAP; George et al., 1999).^1^ Self-report measures—mainly used within the social psychology tradition of attachment research—focus on believes about personal attachment relationships. In contrast, the assessment of attachment representation by the AAI and the AAP, developed within the developmental psychology tradition, is based on the coherence of mind in talking about one’s own attachment history (AAI) and the coherence of narratives elicited through attachment relevant pictures (AAP), respectively. Indeed, associations between explicit and implicit methods of adult attachment are only modest (de Haas et al., 1994; Crowell and Treboux, 1995). Focusing on experiences with primary caregivers and giving details on how emotions in attachment-related situations are regulated by an individual (e.g., Allen and Miga, 2010), the AAI is often marked as the gold standard.

Early childhood appears to be a critical period for the development of the IWM of attachment. At the same time, the first 2 years of life are crucial for the human brains’ structural development. In this developmental period neural circuits become organized and adjusted to the child’s environment (Rutter, 2002). This is why during the early years these neuronal changes are highly affected by experiences with the attachment figure (Luby et al., 2012; Whittle et al., 2015). In adulthood, cross-sectional studies have shown that individuals with different attachment qualities, presumably resulting from attachment experiences during early development, differ regarding the neural processing of emotional information as assessed by brain imaging and EEG measures (e.g., Schore, 1994; Vrtička et al., 2008; Zhang et al., 2008; Suslow et al., 2009; Dan and Raz, 2012; Leyh et al., 2016; for a review, also see Gander and Buchheim, 2015). For example, using a self-report measure Vrtička et al. (2008) found avoidant attachment, associated with low emotional availability of the attachment figure, to be related to lower responses of the primary somatosensory cortex to masked sad faces. The authors interpreted the finding as a habitual unwillingness to deal with a partner’s distress and his or her needs for proximity. Furthermore, anxious attachment was related to a left amygdala response evoked by angry faces when associated with negative feedback. Several studies indicate that at an automatic processing level individuals with anxious attachment quality are more responsive to emotional facial signals than securely attached individuals (Vrtička et al., 2008; Donges et al., 2012).

Neurophysiological activation underlying social-emotional processes can best be investigated by using event-related potentials (ERPs) because of their high temporal resolution. Currently available ERP studies provide evidence of attachment-related differences in neuronal processing of facial emotions (Zhang et al., 2008; Fraedrich et al., 2010; Escobar et al., 2013; Leyh et al., 2016). While early ERP responses usually index bottom-up sensory mechanisms, which are sensitive to stimuli characteristics and therefore indicate pre-attentional processes, top-down control mechanisms occur at later processing stage and reflect in mid-latency ERP responses (>300 ms) interpreted as correlates of attention allocation, arousal, control and/or awareness (Polich, 2007). There is convincing evidence that later ERP components are modulated by attachment representation. According to Zhang et al. (2008), avoidantly attached subjects, in response to emotional facial expressions, again had lower N400, which is negative going component associated with semantic integration (Kutas and Federmeier, 2009). Moreover, evidence suggests that mothers with insecure attachment representation show lower P3 amplitudes than securely attached mothers after presentation of infant emotion pictures (Fraedrich et al., 2010; Leyh et al., 2016). The P3 component reflects task characteristics like cognitive demand (Isreal et al., 1980) and task difficulty (Polich, 1987). It is influenced by stimulus novelty (Polich, 2007) as well as subjects’ arousal level (Kahneman, 1973). The underlying processes are thought to be the inhibition of extraneous neural processes associated with attention allocation and memory (Polich, 2007). In combination with imaging studies it can be assumed that the dampened neural response to infant emotion pictures reported above indicates deficits in perception of and responsiveness to emotional stimuli in insecure-dismissing subjects. However, it is still unclear how neural processing is altered in insecure-preoccupied attached subjects. It remains open whether restrictions in neurophysiological processing are also given in subjects with an insecure-preoccupied attachment representation, as due to the small group sizes this group frequently has neither been included in systematic analyses nor has it been combined with the insecure-dismissing group to form a group of insecure subjects (e.g., Leyh et al., 2016).

Taken together, these findings suggest that experimental paradigms targeting the processing of social-emotional stimuli are capable of activating the attachment system as they have repeatedly shown to reveal attachment related differences at both the behavioral as well as the neurophysiological level. From an attachment theory perspective this makes perfect sense, as especially situations triggering negative emotions with a need for emotional regulation are believed to activate the attachment system, hence, shedding light on individual strategies to handle these emotions. However, the studies reported above have solely focused on altered processing of emotional information with regard to attachment rather than looking at how emotional contexts affect individual processing of neutral information. Indeed, such knowledge would be highly relevant for understanding an individual’s functioning in everyday life in the face of emotional stressors. Addressing this void in the literature the main objective of the current study was to investigate whether emotional contexts influence the neuronal processing of neutral (non-emotional) information depending on attachment.

The studies reported above provide evidence on the influence of attachment qualities on the processing of emotional information which can be expected on the base of assumptions guided by attachment theory. Based on these findings it can be assumed that effects of attachment quality on cognitive processing are also evident when challenging subjects’ capacities of emotional regulation by embedding neutral task-relevant information in emotional (especially negative) contexts. In insecure subjects inefficient attempts to regulate negative emotions provoked by a negative emotional context may lead to an enhanced allocation of cognitive resources to the processing of the task-irrelevant emotional information. Consequently, there may be a lack of cognitive capacities needed to effectively process task-relevant information.

Regardless of attachment, Meinhardt and Pekrun (2003) investigated the impact of emotions on attentional resource allocation in an ERP-experiment by combining auditory and visual stimulation. They examined the P3 in an oddball task using auditory stimuli during presentation of positive, negative or neutral emotional pictures or by imagination of emotional events. They found that the P3 amplitude to auditory stimuli was reduced during emotional compared to neutral conditions. This supports that emotional states influence cognitive processing. Furthermore, Albert et al. (2010) showed the impact of emotional contexts on response inhibition to neutral stimuli in an ERP-experiment using a Go/No-go task. They presented two different letters (as Go and No-go-stimuli) on the background of positive, negative and neutral pictures, and found a modulating effect of emotional contexts on the no-go P3. Larger frontocentral no-go P3 amplitudes and stronger anterior cingulate cortex activation were found to stimuli that required withholding a pre-potent response during positive contexts compared to negative contexts. This shows on a neuronal level that more inhibitory control is necessary to withhold a pre-potent response in positive contexts.

As differences in the quality of attachment are associated with the ability to efficiently regulate emotions, attachment representation might, consequently, be an important moderator between task-irrelevant emotional contexts and the processing of neutral, task-relevant information. Thus, the current study aimed to test this assumption by investigating the role of attachment on cognitive performance in emotional and neutral contexts as indexed by the ERP- component P3, using an oddball paradigm.

Securely attached individuals are assumed to be more capable to effectively regulate emotional states, expectably enabling them to dedicate more cognitive resources to a given task. By theory, a securely attached individual’s regulatory capability especially comes to light when dealing with negative emotions. Consequently, the impact of negative emotional contexts on task relevant processing of embedded neutral information might be less intense. Thus, we hypothesized that especially negative emotional contexts reduce P3 amplitudes during information processing in insecure-dismissing and presumably insecure-preoccupied attached individuals, compared to securely attached individuals.

## Materials and Methods

### Participants

The sample consisted of 42 right-handed young adults (22 female, 20 male) ranging from 17 to 22 years (*M* = 19.46, *SD* = 1.27). One and five of the participants have graduated from secondary school after 9 years (low level) and 10 years (medium level). Twelve participants were striving for a high level graduation (after 12 or 13 years), 24 already had graduated on this level. One participant dropped out before the completion of the study. Thus, data from 41 individuals was used for statistical analysis. Participants were recruited with flyers to take part in a larger overall study, which included three laboratory visits and they were compensated 40 € in total.

### Measures

The current article refers to data collected at the first and third laboratory assessment. At the first laboratory visit attachment representation (AAI; George et al., 1985) and handedness (EHI; Oldfield, 1971) were assessed. Right-handedness was confirmed in all individuals. Within a few weeks, there were two further laboratory assessments to collect neurophysiological data using various paradigms. The current article refers to the investigation of relations between attachment representations and ERP data assessed during the final laboratory visit.^2^

#### Adult Attachment Interview

Attachment representation was assessed by the AAI (George et al., 1985), a semi-structured interview focusing on significant childhood experiences, attachment relevant situations in childhood, the evaluation of these experiences as well as the current relationship to the primary caregivers. Transcripts of these interviews were coded on the basis of Main et al. (2002) manual. The judgment of the narrative coherence, idealization and derogation of parents and/or attachment, as well as current preoccupying anger and passivity of speech results in one of the three main attachment categories: Secure (F), Insecure-Dismissing (Ds), Insecure-Preoccupied (E). The AAI’s reliability and validity is well established (for a review, see Gloger-Tippelt, 2001).

In the present study, the German translation of the original English AAI protocol was used (Gloger-Tippelt, 2001). The AAIs were conducted by the third author and a psychology student after receiving extensive training. Interviews were audio-taped, transcribed and all personal information about the participants was removed from the transcripts. The transcripts were coded by a certified coder.^3^ To test reliability 10 randomly selected AAIs were coded by a second certified coder.^4^ Coding agreement was 90% (*κ* = 0.84, *p* ≤ 0.001).

#### ERP Experiment

##### Stimuli

Oddball stimuli consisted of one out of two white letters, a frequently shown “M” (80%; standard stimulus) and a rarely shown “W” (20%; target stimulus). To increase perceptibility they were shown against a black square sized 2.7 cm × 2.7 cm. Nine pictures taken from the International Affective Picture System (IAPS; Lang et al., 2008) were used as context pictures. They were sized 38.6 cm × 29 cm filling the whole visual background of the 19” screen. Background pictures were presented with subtending 10.2° by 7.8° of visual angle, oddball stimuli with 0.45° by 0.45° of visual angle. Background context pictures were selected from the IAPS on the basis of normative valence ratings (vr). Selection criteria were as follows: negative: vr < 1.98 (PR = 05); neutral: 4.7 ≤ vr ≤ 5.3; negative: vr < 7.57 (PR = 95). The range of the vr of the pictures used was between 1.52 and 1.80 for negative pictures (IAPS No. 3015, 3060, and 3530), between 5.12 and 5.33 for neutral pictures (IAPS No. 2745.1, 5530, and 7493), and between 8.24 and 8.59 for positive pictures (IAPS No. 1710, 2058, and 2165). The mean arousal ratings of the pictures varied as expected, with highest arousal for negative pictures (5.90 to 7.12) and lowest for neutral ones (2.87 to 3.41) and in-between values for positive pictures (5.05 to 5.31). Between conditions, background pictures were matched in mean luminance and spatial frequency using parameter values provided by Delplanque et al. (2007). Mean luminance for positive, neutral and negative pictures were 95, 97, and 113, respectively. There was no significant difference among them (*F*_(2,6)_ = 0.46, ns). Differences regarding the spatial frequency were tested for nine frequency bands (from < 2 to 256–512 cycles per image) and each type of layer (grayscale, red, green, blue) in a multivariate analysis of variance (MANOVA). There was no effect for valence (*F* < 1; ns).

##### Procedure

During the ERP experiment, participants were seated in a dimly lit, electrical and acoustically shielded cabin in a comfortable chair. The monitor presenting the stimuli was placed in a viewing distance of 115 cm. Prior to the oddball experiment participants were instructed to press a button as fast as possible when detecting the target stimulus (“W”). The task was performed during three emotional context conditions (negative, positive, neutral) that were generated by IAPS stimuli appearing on the screen as background pictures. Emotional context conditions were presented in randomized order and consisted of 390 trials (312 standard/78 target) per condition. Within each emotional context condition there were three blocks (130 trials) each presenting one of three negative, positive, or neutral background pictures, respectively. Each block started with the presentation of three additional standard trials. The order of background pictures within each condition was randomized across subjects. During each block the respective context background picture remained on screen. In every single trial oddball stimuli appeared at the center of the screen against the respective background picture, thus, being embedded in it. This simultaneous presentation of stimuli and background pictures remained on screen for 200 ms followed by an inter-stimulus interval of 1300 ms, during which the oddball stimuli, the white letter, disappeared. After each block participants were asked to push a button to continue the experiment.

The stimulus presentation was controlled by the experimental software Inquisit (Millisecond Software, Seattle, WA, USA). The behavioral responses following standard and target stimuli (button press) were registered in ms after stimulus onset. Correct responses were defined as button press after target presentation.

##### Data Recording and Analysis

EEG was recorded according to the international 10–20 system with active electrodes based on high-quality Ag/AgCl sensors 5 mm in diameter from 60 electrode sites^5^ while keeping impedances below 25 kΩ. To assess eye movements additional electrodes were placed below and above the left eye, as well as next to the outer canthi. The ground electrode was placed at AFz and data was referenced to activity recorded from electrode site FCz. Signals were acquired using BrainAmp Standard amplifier (Brain Products, Gilching, Germany) allowing for the recording of frequencies ranging from 0.016 to 1000 Hz with a resolution of 0.1 μV per bit and a measurement range of ±3.28 mV. Sampling rate for all channels was set to 500 Hz and signals were digitized using a 16 bit A/D converter. Recording and analyzing of the EEG was performed using BrainVision Software (Brain Products, Gilching, Germany).

During offline-processing the EEG signal was re-referenced to the average of the mastoids and digitally filtered using a 0.1 Hz high pass and a 30 Hz low pass filter with a 24 dB/oct gradient. The EEG signal then was segmented into epochs ranging from 200 ms pre- to 700 ms after stimulus onset. The pre-stimulus window of 200 ms was used for baseline-correction. To correct for saccadic eye movements or eye blinks the Gratton and Cole Procedure was applied (Gratton et al., 1983). Further artifacts were removed semi-automatically allowing for voltage steps of 50 μV between sampling points and a voltage threshold of ±70 μV.

Finally, standard and target stimuli segments were averaged for each participant and condition. Based on previous literature and a visual inspection of the grand average waveforms, the P3 was scored as the maximum positive peak between 330 and 600 ms after stimulus onset at midline electrodes Fz, Cz, Pz. With brain activity decreasing from parietal to frontal scalp it was found to show typical scalp topography (Johnson, 1993). In addition, to test whether the effects are consistent and not restrict to the midline channel, lateral electrodes on the parietal site were also included (P3, P1, P2, and P4). Number of correct responses and response latencies (ms) of behavioral responses to target and standard stimuli were stored by the experimental software.

### Statistical Analysis

Number of correct responses and reaction times to targets as well as false alarms were analyzed by a two-way MANOVA with a repeated measure factor for context and an independent factor for attachment representation.

To examine the impact of attachment representation on P3 amplitude responses repeated measure MANOVAs were conducted with repeated measure factors for electrode (midline: Fz, Cz, Pz; parietal: P3, P1, Pz, P2, P4), stimulus type (standard vs. target), context (negative, neutral, positive) and an independent factor for attachment representation (secure, insecure-dismissing, insecure-preoccupied). The main focus of analysis was on effects concerning attachment, hence only significant effects including attachment security are reported in detail. Attachment relevant effects were further analyzed *post hoc* using LSD tests. Due to the small sample size of the insecure-preoccupied group, analyses of variances were additionally tested without this group, hereby restricting the analysis on a comparison of the secure and insecure dismissing group.

## Results

### Attachment Representation

The scoring of the AAIs resulted in the following distribution of attachment representations: There were 22 persons with a secure attachment representation and 19 with an insecure one, among the latter 14 persons had an insecure-dismissing and 5 persons had an insecure-preoccupied attachment representation.

Preliminary analyses showed that attachment representation was not associated with the subjects’ age and education (*F*_(2,39)_ < 1, ns). Mean ages were 19.4, 19.6 and 19.4 years for the secure, the insecure-dismissing and the insecure-preoccupied group, respectively. The respective levels of education (on a four-point scale) were 3.5, 3.4, and 3.4. However, there was a significant association between attachment and gender (*χ*^2^ = 10.1, *p* < 0.01). A nearer inspection of the data showed that boys were more frequently found in the insecure-dismissing (11 of 14) and less frequently in secure pattern (6 of 16), while there was no difference within the insecure-preoccupied group (two boys, three girls). Therefore, gender was used as a covariate in the analyses regarding attachment.

### Behavioral Data

**Figure 1:**
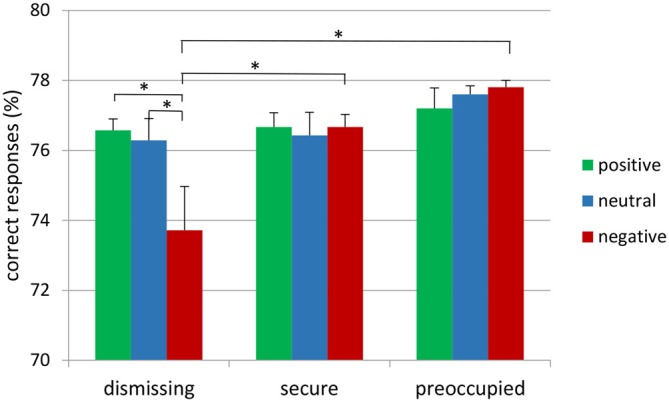
Frequency of correct responses to target stimulus presented on the background of negative, neutral and positive background stimuli in secure, insecure-dismissing and insecure-preoccupied subjects (asterisks indicate significant differences, *p < 0.05).

The attachment × emotional context repeated measure MANOVA for correct target responses with gender as a covariate did not show main effects, but resulted in a significant interaction between emotional context and attachment representation (*F*_(4,72)_ = 2.65, *p* ≤ 0.05, *η*^2^ = 0.13; see Figure 1). This interaction was also found after exclusion of the insecure-preoccupied group (*F*_(2,64)_ = 4.04, *p* < 0.05, *η*^2^ = 0.11). LSD *post hoc* tests (*p* < 0.05) showed that effects of emotional context were only given for insecure-dismissing subjects. These subjects detected fewer targets in the negative emotional context than in neutral or positive contexts (see Figure 1). In addition, the number of correct target responses in the negative emotional context was lower in the insecure-dismissing group than each of the other groups. There were no significant effects for false alarms and response times to target (for the means, see Table 1).

**Table 1:** **Frequency of correct responses and false alarms as well as response times depending on emotional context and attachment representation**.

	Emotional context		
	Positive	Neutral	Negative
**Correct responses**
Secure	76.7 (1.6)	76.4 (3.0)	76.7 (1.7)
Insecure-dismissing	76.6 (1.2)	76.3 (2.3)	73.7 (4.7)
Insecure-preoccupied	77.2 (1.2)	77.6 (0.5)	77.8 (0.5)
**False alarms**
Secure	1.2 (1.7)	1.7 (2.1)	1.0 (1.6)
Insecure-dismissing	0.9 (1.3)	1.5 (1.4)	1.9 (1.9)
Insecure-preoccupied	0.2 (0.4)	0.6 (1.3)	1.2 (2.7)
**Response time**
Secure	507.5 (57.3)	520.3 (66.3)	511.3 (58.8)
Insecure-dismissing	502.6 (61.2)	506.7 (58.6)	519.7 (53.2)
Insecure-preoccupied	480.6 (34.1)	499.8 (26.2)	495.2 (42.4)

*Means and standard deviations*.

### P3

**Figure 2:**
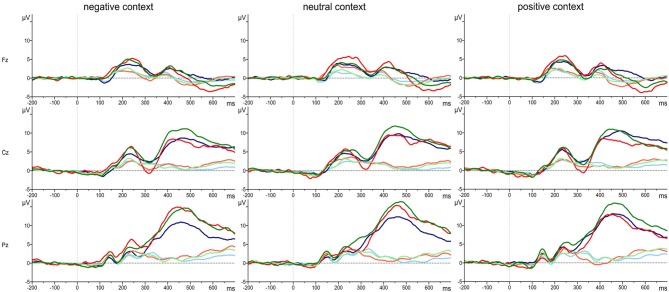
**Grand average waveforms of P3 responses to target stimuli (dark colored lines) and standard stimuli (light colored lines) in positive, neutral and negative emotional context for Fz, Cz and Pz in persons with a secure (green line), insecure-dismissing (blue line) or insecure-preoccupied attachment representation (red line)**.

**Figure 3:**
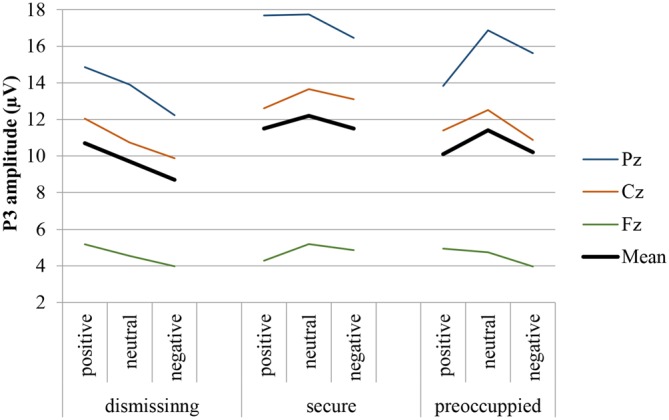
**P3 response (Fz, Cz, Pz, and mean) to target stimulus presented on the background of negative, neutral and positive stimuli in secure, insecure-dismissing and insecure-preoccupied subjects**.

The grand average waveforms of the P3 responses of the three groups of attachment representations are shown in Figure 2 for each of the three electrode positions (Fz, Cz, Pz) and separately for positive, neutral and negative emotional context. Comparing the responses to the target and standard stimuli the grand means show the typical response pattern of an oddball paradigm with the typical P3 deflection for the target stimuli. In addition, Figure 3 clearly indicates differences in the P3 deflection between the attachment groups.

The four-way repeated measure MANOVA for P3 amplitude responses with the factors electrodes (midline: Pz, Cz, Fz), context, stimulus type, and attachment and with gender as a covariate resulted in a significant main effect for electrode (*F*_(2,74)_ = 12.87, *p* ≤ 0.001, *η*^2^ = 0.26) stimulus type (*F*_(1,37)_ = 11.95, *p* ≤ 0.001, *η*^2^ = 0.24), qualified by a significant two-way interaction between electrode and stimulus type (*F*_(2,74)_ = 16.61, *p* ≤ 0.001, *η*^2^ = 0.31) and by a significant three-way interaction between context, stimulus type and attachment (*F*_(4,74)_ = 2.72, *p* ≤ 0.05, *η*^2^ = 0.13). This three-way interaction including attachment remained significant after exclusion of the preoccupied group (*F*_(2,66)_ = 3.54, *p* ≤ 0.05, *η*^2^ = 0.10). The first three effects including stimulus type and electrode effect (higher amplitudes for target stimuli, increasing amplitudes from Fz to Cz to Pz) depicts the typical P3 effect with an increasing effect from the frontal to the parietal brain area. Regarding the three-way interaction between stimulus type, emotional context, and attachment, *post hoc* multiple LSD comparisons revealed an effect of emotional context only in the insecure-dismissing group in which the P3 amplitude was significantly lower in the negative emotional context than in the positive emotional context. In addition, the P3 amplitudes of the insecure-dismissing group in the negative emotional context were significantly lower than the amplitudes of the secure group.

The four-way repeated measure MANOVA for P3 amplitude responses with the factors electrodes (parietal: P3, P1, Pz, P2, P4), context, stimulus type, and attachment and with gender as a covariate resulted in a significant main effect for electrode (*F*_(4,148)_ = 3.45, *p* ≤ 0.01, *η*^2^ = 0.09) stimulus type (*F*_(1,37)_ = 26.26, *p* ≤ 0.001, *η*^2^ = 0.42), qualified by a significant two-way interaction between electrode and stimulus type (*F*_(4,148)_ = 4.48, *p* ≤ 0.01, *η*^2^ = 0.11) and by a significant three-way interaction between context, stimulus type and attachment (*F*_(4,148)_ = 2.57, *p* ≤ 0.05, *η*^2^ = 0.12). The first three effects including stimulus type and electrode effect depicts the typical P3 effect regarding central and lateral brain areas with higher amplitudes for target stimuli with the highest amplitude at Pz and decreasing amplitudes at lateral sites P1 and P2 and still more decreasing at P3 and P4 (see Table 2). Regarding the three-way interaction between stimulus type, emotional context, and attachment, *post hoc* multiple LSD comparisons revealed an effect of emotional context only in the insecure-dismissing group in which the P3 amplitude was significantly lower in the negative emotional context than in the neutral and positive emotional context. In addition, the P3 amplitudes of the insecure-dismissing group in the negative and neutral emotional context were significantly lower than the amplitudes of the secure group.

**Table 2:** **P3 amplitudes (μV) to target stimuli depending on emotional context and attachment representation**.

	Insecure-dismissing			Secure			Insecure-preoccupied		
	Positive	Neutral	Negative	Positive	Neutral	Negative	Positive	Neutral	Negative
Fc	5.18	4.55	3.98	4.28	5.19	4.87	4.95	4.75	3.96
Cz	12.04	10.73	9.87	12.61	13.66	13.10	11.40	12.51	10.88
Pz	14.86	13.90	12.24	17.68	17.75	16.47	13.83	16.88	15.63
P1	13.45	12.53	10.77	15.71	15.48	14.74	13.55	16.08	15.74
P2	13.94	12.88	11.21	17.27	16.46	15.94	11.91	14.39	13.72
P3	10.69	10.16	8.61	14.64	14.09	13.36	11.13	13.01	12.15
P4	11.60	10.29	9.15	13.85	13.96	12.90	9.85	11.60	11.40

After exclusion of the preoccupied group the three-way interaction between emotional context, stimulus type, and attachment failed to reach significance. Instead there was a main effect of attachment (*F*_(1,33)_ = 5.48, *p* ≤ 0.05, *η*^2^ = 0.14) qualified by a two-way interaction between stimulus type and attachment (*F*_(1, 33)_ = 4.55, *p* ≤ 0.05, *η*^2^ = 0.12). As can be seen from Table 2, the P3 amplitude in response to target is higher in secure than in insecure-dismissing subjects.

## Discussion

The current study aimed to link attachment representation to cognitive and emotional information processing on a behavioral and neurophysiological level. At the behavioral level, subjects with insecure-dismissing attachment representation responded less frequently with correct responses to targets in negative contexts than securely or insecure-preoccupied attached subjects, which indicates that they recognized fewer target stimuli. On a neurophysiological level, ERP-data showed that subjects with insecure-dismissing attachment representation had smaller P3 amplitudes to target stimuli when they were embedded within a negative as compared to a neutral and positive context, which was not the case in the secure and insecure-preoccupied attachment groups. These findings support the hypothesis that attachment representation moderates the association between emotion and information processing.

### Behavioral Results

In the present study, negative emotional context reduced hit rates to neutral stimuli in insecure-dismissing compared to securely attached subjects. Similar findings have been reported in several studies showing restrictions in processing negative facial expressions shown by avoidantly attached subjects as assessed by self-report questionnaire. For example, Dan and Raz (2012) investigated responses to angry and neutral faces in subjects with different attachment qualities. They reported slower response times in trials with angry faces as compared to trials with neutral faces in subjects with an avoidant attachment. These differences were not found in the secure or anxious attachment groups. Similarly, Escobar et al. (2013) reported slower reaction times to stimuli with negative valence in insecurely attached adolescents as compared to securely attached ones. Effects on both response times and correct answers were reported by Fraedrich et al. (2010) who reported shorter response times after presentation of negative faces in secure as compared to insecure-dismissing mothers and a smaller amount of false alarms (i.e., less errors) in secure mothers.

Although the present study did not replicate the findings regarding response times, the fewer correct answers in insecure-dismissing subjects as compared to the other attachment groups depicts a restricted processing ability in the context of negative emotion. Presumably the present stimulus, either “W” or “M” may invite for faster responses with the side-effect of a higher error-rate, while decisions on facial valence are more complex. Consequently, subjects take more time but have lower error-rates.

These findings are in line with theoretical assumptions from attachment theory postulating deficits in emotional regulation abilities in insecure-dismissing individuals in distressing or threatening situations, in which the attachment system is activated. This is explained by the child’s experiences with the caregiver. While sensitive responding to the infant’s emotional expression and emotional needs in mothers facilitates the development of emotional competences and finally contributes to the development of a secure infant-mother attachment, infants of insensitive or rejecting mothers are restricted in the development of emotional regulation strategies, their ability recognize emotions, to express and communicate their needs and to refer to the caregiver as a source of emotional support. For example, a restricted ability in emotion recognition in insecure-dismissing subjects was also found in behavioral studies with children or adolescents (e.g., Spangler and Zimmermann, 1999; Steele et al., 2008). During the AAI, insecure-dismissing individuals lack to produce coherent answers when confronted with questions regarding emotional experiences during their childhood. This seems to indicate restricted processing of emotional information (Main et al., 2002).

### P3 Results

First of all, the pattern of the P3 responses along the midline (frontal, central and parietal) demonstrating decreasing P3 amplitude responses from frontal to central to parietal areas as well as the pattern of the P3 amplitude responses along the parietal line (central and lateral) demonstrating higher central and lower lateral amplitudes were as expected from research literature and verify the validity of the assessment.

In the current study, attachment groups differed significantly with regard to the P3 ERP component. Individuals with an insecure-dismissing attachment representation showed significantly smaller P3 amplitudes to target stimuli when embedded within negative as compared to neutral or positive emotional contexts. In addition, the P3 amplitudes of insecure-dismissing subjects during negative contexts were smaller than those of secure subjects. This pattern was found independent of the location of the electrodes included, both for the amplitudes along the midline (Pz, Cz, Fz) and the amplitudes along the parietal line (P3, P1, Pz, P2, P4).

According to Polich (2007) the P3 amplitude is hypothesized to index allocation of attentional resources. Thus, the P3 amplitude typically increases for infrequently appearing oddball stimuli. This is especially true when task conditions are undemanding. However, an increase in task requirements appears to occupy cognitive resources leading to smaller P3 amplitude responses (Polich, 1987; Kok, 2001). Similarly, evidence suggests that events independent of the oddball task (i.e., engagement in a secondary task) draw upon attentional ressources resulting in smaller P3 amplitude responses (e.g., Isreal et al., 1980).

Transferring the attention allocation theory of Polich (2007) to the present study, it can be concluded that negative emotional contexts drain on information processing in insecure-dismissing individuals. Thus, the smaller amplitudes in the P3 of insecure-dismissing subjects in negative contexts in our study may reflect lower engagement in target stimulus processing. It can be assumed that top-down processes modulating the P3 come along with a concurrent neurophysiological activation that suppresses target detection in negative contexts (also see Vrtička et al., 2008). Indeed, as reported above, few studies including the current paper found insecure-dismissing (or avoidant) subjects to show behavioral deficits associated with stimuli of negative valence as well (e.g., Dan and Raz, 2012; Escobar et al., 2013). This interpretation is supported by the above reported behavioral deficits of insecure-dismissing attached individuals in recognizing targets embedded within a negative context. From this perspective, the reported neurophysiological processes involved in the processing of task-irrelevant information might contribute to attachment related differences in behavioral target detection within negative contexts.

Similarly, differences between insecure-dismissing and secure individuals in the P3 amplitude in response to faces of different valence were also found by Fraedrich et al. (2010) in a study with mothers looking at positive, neutral and negative infant faces. In addition, Leyh et al. (2016) found an elevated P3 positivity during perception of infant emotional faces in securely compared to insecure mothers. More precisely, they found higher P3 amplitudes in secure mothers when asked to focus on positive or negative faces and when asked to differentiate neutral faces from negative faces. According to Polich (2007) this indicates that insecure mothers allocated fewer attentional resources to recognize emotional faces, which could be interpreted as a defensive effort in light of emotional information. The heightened allocation of attentional resources found in secure as compared to insecure mothers may explain why securely attached mothers are more sensitive to their infants’ signals of emotional needs. The main difference between the study of Fraedrich et al. (2010) and the current study was that in the former one, subjects were asked to draw attention to emotional expressions while in the current study attention had to be focused on a neutral stimulus while ignoring the emotional context. Nevertheless, there were similar results indicating reduced P3 responses associated with negative emotions in insecure-dismissing subjects.

The findings of other studies were somewhat different to the findings of this study. Zhang et al. (2008) did not find associations between attachment quality and the P3 amplitude in general. Only avoidant attachment was associated with lower P3 amplitude responses and a later component, namely the N400, associated with semantic integration. Mark et al. (2012) investigated ERP responses to sad and angry faces depending on dimensions of secure, anxious and avoidant attachment. They did not assess group comparisons between secure and insecure attachment, but calculated associations between ERP responses and the attachment scales. While their findings did not suggest any association between facial emotional processing and avoidant attachment, P3 amplitude was positively associated with anxious attachment and negatively with secure attachment. These effects, however, did not reach significance after controlling for trait anxiety.

A reason for the different findings of Zhang et al. (2008) as well as Mark et al. (2012) may be the attachment assessment procedure, as these two studies—in contrast to the current study as well as the studies by Fraedrich et al. (2010) and Leyh et al. (2016)—used self-report measures.

In sum, interpreting the current findings of insecure-dismissing subjects’ dampened P3 amplitude responses to target stimuli in negative contexts in terms of reduced attentional processing capacities well fits with the behavioral findings in the current study. More precisely, this study suggests that the insecure-dismissing group may allocate more attention resources to task-irrelevant negative emotional information, presumably in a defensive regulatory effort, which may in turn contribute to poorer task performance at a behavioral level. This interpretation is supported by previous studies cited above reporting restrictions in the ability to recognize and to regulate negative emotions in infants and adolescents with a history of insecure attachment relationships.

While securely attached subjects significantly differed in neural processing from subjects with an insecure-dismissing attachment relationship, we did not find differential processing patterns when comparing secure and insecure-preoccupied subjects. There are several explanations for this non-finding. Firstly, due to the very different modes of emotional regulation associated with the two different insecure attachment patterns and also due to the explanation of their determinants (Cassidy and Berlin, 1994) similar neurophysiological responses would not be expected anyway. For example, Mark et al. (2012) found an association between the P3 amplitude and anxious attachment, but not with avoidant attachment.

Secondly, from a methodological perspective the relatively low number of insecure-preoccupied subjects in our study (which could be expected with respect to known normative distribution of attachment patterns) may have resulted in low statistical power, which makes further neurophysiological studies of this attachment subgroup necessary. Moreover, data inspection revealed that the mean scores of the insecure-preoccupied subjects actually lie between the secure and insecure-dismissing ones, with being more similar to the secure subjects.

Due to the small number of preoccupied subjects we, additionally, calculated the analyses of variance after exclusion of the preoccupied group. In most cases, the findings regarding attachment representation remained essentially the same. Only for the ERP analyses along the parietal line, the interaction between emotional context and attachment for the target stimuli disappeared, and a main effect of attachment in response to target stimuli was found indicating that insecure subjects allocated fewer attentional resources not only in a negative emotional context, but in general. This is in line with Fraedrich et al. (2010) who found a smaller P3 response in dismissing mothers to infant faces of any valence. It may be that in dismissing subjects the processing of social or emotional information during an oddball task requires additional resources, in general, which decreases available attentional resources in this attachment group, resulting in decreased P3 amplitudes. From this perspective the supposed modulating effect of attachment might already be present at an earlier stage of regulation regarding emotional tension induced by the experimental task *per se*.

In summary, the present study shows that the attachment representation influences neurophysiological processing of task-relevant stimuli embedded within emotional contexts. This finding provides further empirical evidence for the assumption that the inner working model of attachment influences perception, processing and interpretation of emotional cues (Spangler and Zimmermann, 1999). As the assessment of attachment by the AAI focuses on experiences with the primary caregiver our findings suggest that the early attachment relationship or the attachment history with the caregiver has an impact on neurophysiological processing of emotional information. Support comes from few studies, presenting results that early interaction with the primary caregiver affect the development of brain structures significantly (Rutter, 2002; Luby et al., 2012).

Furthermore, this study combines a neurophysiological approach with the assessment of attachment on a representational level, in contrast to other studies (Zhang et al., 2008; Dan and Raz, 2012; Mark et al., 2012) that assessed attachment style by self-report questionnaires. This further shows that associations between attachment and neurophysiological processes may depend on the measure used for assessment of attachment. The AAI assesses attachment qualities implicitly on a representational level. Thereby, it focuses on experiences with primary caregivers and gives details on how an individual regulates its emotions regarding attachment related situations (e.g., Allen and Miga, 2010). Using the AAI may be the most effective way to gain information about emotion regulation in early childhood retrospectively. Indeed, this developmental phase is especially sensitive to neurophysiological alterations induced by parenting behavior as the brain undergoes many plastic changes. To further study the developmental trajectory of attachment development associated with neuronal insights later in life, the AAI seems to be relevant because of its developmental implications. Future research could also add behavioral assessments to investigate the generalizability of the effects to daily social interactions.

The present study investigated neural processing of neutral targets in emotional contexts to study how attachment moderates the link between processing of task-irrelevant stimuli in the environment (here: the emotional context) and task relevant information processing. In contrast, most other studies focus on facial expressions to investigate emotion perception in others (Green et al., 2007; Milanak and Berenbaum, 2014). This limits comparisons to the effects of other studies on attachment and emotion processing. Moreover, in the present study, pictures showing emotional contexts did not necessarily include socially relevant situations. The question remains, if effects became stronger when real social situations or even better a live social interaction would be presented. This also assigns to the neutral stimuli “W” and “M” which usually do not have any significant meaning in our daily life. While the focus of the study was to investigate the impact of emotional surroundings on neutral targets, the ecological validity should be enhanced for further studies by using socially relevant situations as context and more relevant objects, like familiar persons or objects with emotional relevance as targets.

### Limitations

Some limitations have to be considered in this study. First, the sample size of the study is limited, and second, the sample size of the different attachment groups varies considerably. Specifically, the insecure-preoccupied group is rather small. It should be noted that the distribution found in this study corresponds with the typical distribution found in several studies (for e meta-analysis, see van IJzendoorn, 1995). This may suggest using non-parametric procedures for hypothesis testing. However, using nonparametric procedures would restrict possibilities for multifactorial designs. Therefore, we nevertheless used parametric procedures. Although the insecure-preoccupied groups is quite small (*n* = 5), we nevertheless included this group into the study and into the statistical analyses in order to provide at least the descriptive information about this group for the interested reader. As the main findings of this study mainly refer to differences between the insecure-dismissing and the secure group, we did not make specific conclusions regarding the insecure-preoccupied group. Moreover additional analyses without the insecure-preoccupied groups resulted in the same effects.

## Conclusion

Our findings support assumptions about moderation of emotional information processing by differences in attachment representation. Specifically it has been shown that negative emotional information restricts cognitive emotional processing in insecure-dismissing subjects. This indicates that it is sufficient to alter context information to influence neural processing in insecure-dismissing subjects in contrast to varying emotional content of stimuli in the focus of attention. However, in the case of insecure-preoccupied subjects it remains vague to which extent neural processing is altered by varying emotional contexts.

## Ethics Statement

The study was conducted in accordance with the Code of Ethics of the German Psychological Association (from 09/28/2004), which is essentially based on the Code of Ethics of the APA (Ethical Principles of Psychologists and Code of Conduct, American Psychologist, 2002, 57, 1060-1073). According to the rules of the German Research Foundation, it was not required to apply for a formal vote for this study; because (1) the participants were healthy (no patient groups); (2) we did not use invasive methods; and (3) there participation did not provide any risk to the subjects. Finally (4), for studies using ERP assessments, a formal vote only is required if the subjects are younger than 14 years and older than 65 years.

### Informed Consent

The participants were informed about the aims of the study and about the methods used. In addition, they were informed that (1) their participation was voluntary and that they at any point of time had the right to withdraw the participation and (2) that the data were treated according to the data protection law, and that they were saved anonymously. Each participant signed the informed consent form before participation.

## Author Contributions

RL: concept, design, collection, analysis and interpretation of data, literature research, writing. CH: analysis and interpretation of data, literature research writing, critical review. MTK: collection, analysis and interpretation of data, writing, critical review. GS: concept, design, supervision, writing, critical review.

## Funding

The study was funded by ressources of the University of Erlangen-Nuremberg.

## Conflict of Interest Statement

The authors declare that the research was conducted in the absence of any commercial or financial relationships that could be construed as a potential conflict of interest.

## Footnotes

^1^To distinguish between explicitly and implicitly assessed attachment categories, we used the different terminology that commonly applies to both approaches: avoidant (explicit measures) vs. insecure-dismissing (implicit measure).

^2^During the second laboratory assessment further EEG experiments were conducted to investigate hemispheric asymmetries (see also Kungl et al., under review) and facial emotion processing.

^3^Johanna Behringer, Institute of Psychology, University of Erlangen-Nuremberg, Germany.

^4^Kathrin Beck, Institute fuer Therapieforschung, Munich, Germany.

^5^F7, F3, Fz, F4, F8, FC5, FC1, FC2, FC6, T7, C3, Cz, C4, T8, TP9, CP5, CP1, CP2, CP6, TP10, P7, P3, Pz, P4, P8, PO9, O1, Oz, O2, PO10, AF4, AF8, F5, F1, F2, F6, FT9, FT7, FC3, FC4, FT8, FT10, C5, C1, C2, C6, TP7, CP3, CPz, CP4, TP8, P5, P1, P2, P6, PO7, PO3, POz, PO4, PO8.
